# Cardiac dysfunction and peri-weaning mortality in malonyl-coenzyme A decarboxylase (MCD) knockout mice as a consequence of restricting substrate plasticity

**DOI:** 10.1016/j.yjmcc.2014.07.008

**Published:** 2014-10

**Authors:** Dunja Aksentijević, Debra J. McAndrew, Anja Karlstädt, Sevasti Zervou, Liam Sebag-Montefiore, Rebecca Cross, Gillian Douglas, Vera Regitz-Zagrosek, Gary D. Lopaschuk, Stefan Neubauer, Craig A. Lygate

**Affiliations:** a Division of Cardiovascular Medicine, Radcliffe Department of Medicine, University of Oxford, UK; b British Heart Foundation Centre for Research Excellence, University of Oxford, UK; c Institute of Gender in Medicine, Charité-Universitätsmedizin Berlin, Berlin, Germany; d Center for Cardiovascular Research, Charité-Universitätsmedizin Berlin, Berlin, Germany; e Mazankowski Alberta Heart Institute, University of Alberta, Edmonton, Alberta, Canada

**Keywords:** α-SA, alpha skeletal actin, Atg3, autophagocytosis associated protein 3, β-MHC, beta myosin heavy chain, CPT-1, carnitine palmitoyl transferase 1, FAO, fatty acid oxidation, GLUT1, glucose transporter 1, GLUT4, glucose transporter 4, HDL, high density lipoprotein, LDH, lactate dehydrogenase, LDL, low density lipoprotein, MCD, malonyl-coenzyme A decarboxylase, MTE-1, mitochondrial thioesterase 1, PDK4, pyruvate dehydrogenase kinase 4, TAN, total adenine nucleotide pool, UCP3, uncoupling protein 3, Metabolism, Fatty acids, Heart failure, Cardiac dysfunction

## Abstract

Inhibition of malonyl-coenzyme A decarboxylase (MCD) shifts metabolism from fatty acid towards glucose oxidation, which has therapeutic potential for obesity and myocardial ischemic injury. However, ~ 40% of patients with MCD deficiency are diagnosed with cardiomyopathy during infancy.

**Aim:**

To clarify the link between MCD deficiency and cardiac dysfunction in early life and to determine the contributing systemic and cardiac metabolic perturbations.

**Methods and results:**

MCD knockout mice (^−/−^) exhibited non-Mendelian genotype ratios (31% fewer MCD^−/−^) with deaths clustered around weaning. Immediately prior to weaning (18 days) MCD^−/−^ mice had lower body weights, elevated body fat, hepatic steatosis and glycogen depletion compared to wild-type littermates. MCD^−/−^ plasma was hyperketonemic, hyperlipidemic, had 60% lower lactate levels and markers of cellular damage were elevated. MCD^−/−^ hearts exhibited hypertrophy, impaired ejection fraction and were energetically compromised (32% lower total adenine nucleotide pool). However differences between WT and MCD^−/−^ converged with age, suggesting that, in surviving MCD^−/−^ mice, early cardiac dysfunction resolves over time. These observations were corroborated by in silico modelling of cardiomyocyte metabolism, which indicated improvement of the MCD^−/−^ metabolic phenotype and improved cardiac efficiency when switched from a high-fat diet (representative of suckling) to a standard post-weaning diet, independent of any developmental changes.

**Conclusions:**

MCD^−/−^ mice consistently exhibited cardiac dysfunction and severe metabolic perturbations while on a high-fat, low carbohydrate diet of maternal milk and these gradually resolved post-weaning. This suggests that dysfunction is a common feature of MCD deficiency during early development, but that severity is dependent on composition of dietary substrates.

## Highlights

• MCD^−/−^ mice exhibit significantly higher early life mortality.
• This is associated with impaired cardiac function, steatosis and energy starvation during suckling.
• The metabolic phenotype and cardiac dysfunction resolve with low-fat post-weaning diet.

## 1 Introduction

The heart has high energy demands that are primarily met by mitochondrial fatty acid oxidation (FAO) [1]. This is regulated at the level of the outer mitochondrial membrane by the activity of carnitine palmitoyl transferase 1 (CPT-1), which acts to shuttle cytosolic long chain acyl-CoA esters into the mitochondria [1]. Malonyl-CoA is a potent endogenous allosteric inhibitor of CPT-1 and thereby a key regulator of fatty acid catabolism. It has a rapid turnover in the heart (t_1/2_ ~ 1.25 min) [2], [3] resulting from the balance between synthesis from acetyl-CoA by acetyl-CoA carboxylase (ACC), and the reverse reaction catalysed by malonyl-CoA decarboxylase (MCD). By controlling malonyl-CoA levels, MCD determines the rate of myocardial fatty acid oxidation [1].

Acute pharmacological MCD inhibition has been shown to decrease fatty acid oxidation and accelerate glucose oxidation in both ex vivo rat and in vivo pig hearts [4], [5], [6]. Furthermore, significant reduction of infarct size has been reported in MCD deficient mice in which glucose oxidation rates were enhanced as a result of the inhibition of fatty acid oxidation [6], [7]. Collectively these findings support the proposition that MCD inhibition can be targeted as an effective approach to treating myocardial ischemia.

While inhibition of MCD and decreasing fatty acid oxidation may have potential therapeutic benefit in the mature heart, it is not clear what consequences of MCD inhibition would be in the newborn period. While foetal hearts rely primarily on glycolysis and lactate oxidation as sources of energy, shortly after birth there is a rapid maturation of fatty acid oxidation [4], [8]. In contrast, glucose oxidation does not mature until weaning [9], and in the newborn period the heart actually becomes more reliant on fatty acid oxidation as an energy source than the adult heart [8], [9]. As a result, MCD inhibition has the potential to decrease cardiac energy production in the newborn period [1].

Concerns for the general safety of MCD inhibition arise from patients with in-born MCD deficiency, a rare autosomal recessive disorder characterized by severe metabolic perturbation in the form of malonic aciduria and variable presentation of developmental delay, seizures, and hypoglycaemia [10], [11].

In particular, cardiomyopathy develops at an early age in up to 40% of MCD deficient patients contributing to morbidity and mortality [12], [13], [14]. However, the pathogenesis of cardiomyopathy remains unclear and may not be directly related to MCD deficiency since not all patients are affected.

The aim of this study was to examine the early cardiac and metabolic phenotypes in MCD knockout (MCD^−/−^) mice to determine whether functional and metabolic alterations are intrinsic features of chronic MCD perturbation during infancy. Here we show that MCD^−/−^ mice universally develop cardiac dysfunction during the peri-weaning period associated with significantly increased mortality and a severe metabolic phenotype of lipid accumulation and carbohydrate depletion, which gradually improves with age. This suggests that restricting substrate plasticity at times of high energy demand and major dietary change is capable of triggering conditions of energy starvation that are detrimental to the young heart.

## 2 Methods

### 2.1 Mouse colonies

MCD knockout mice (MCD^−/−^) were imported from the originating colony at the University of Alberta and backcrossed with C57BL/6J mice for > 6 generations. All mice were generated by heterozygous pair mating thus littermates could be used as the appropriate wild-type controls (MCD^+/+^). Mice were kept under pathogen-free conditions, 12 h light–dark cycle, controlled temperature (20–22 °C), and fed chow and water ad libitum. A breeding diet was used for all dams, LabDiet PicoLab® Mouse Diet 5058, while weaned mice were gradually introduced to PicoLab® Rodent diet 5053 over a 6 week period. This protocol is identical to the originating colony. This investigation conforms to UK Home Office Guidance on the Operation of the Animals (Scientific Procedures) Act, 1986.

### 2.2 In vivo assessment

Mice were imaged and left ventricular (LV) hemodynamic measurements (adult mice) performed as previously described [15], [16]. Body composition was analysed by magnetic resonance relaxometry in conscious restrained mice [15].

### 2.3 Tissue and plasma collection

All mice, knockout (MCD^−/−^), wild type (MCD^+/+^) and heterozygous (MCD^+/−^) were non-fasted, since fasting would have adverse effects at 18 days of age. Mice were killed by cervical dislocation; the heart, skeletal muscle (mixed soleus and gastrocnemius) and liver were excised, snap frozen in liquid nitrogen and stored at − 80 °C for biochemical analyses. Owing to limited tissue availability, body weight and age matched stock C57BL/6J male mice (Harlan, UK) were used as controls for the malonyl-CoA level assessment only. Blood was collected from terminally anaesthetized mice into EDTA microcapillary tubes (Sarstedt, UK) and centrifuged (3000 rpm, 10 min) to obtain plasma. Concentrations of free fatty acids, triglycerides, cholesterol, high-density lipoprotein, low-density lipoprotein, total creatine kinase (CK), lactate, lactate dehydrogenase, and 3-hydroxybutyric acid (ketone bodies) were measured by the Mouse Biochemistry Laboratory, Addenbrooke's Hospital, Cambridge University Hospitals NHS Trust.

### 2.4 Biochemical and gene expression analysis

The activity of the pyruvate dehydrogenase complex (active and total portion of the enzyme), citrate synthase, triglyceride and glycogen content in LV, skeletal muscle and liver were determined spectrophotometrically (Online Supplement Methods). Total creatine, total adenine nucleotide pool (ATP + ADP + AMP) and malonyl-CoA content were measured by HPLC [4], [16]. Total RNA was extracted from heart tissue of MCD^+/+^, ^+/−^, ^−/−^ (n = 4 per group), survivor MCD^−/−^, ^+/+^ (n = 9 per group) and messenger RNA levels analysed using qRT-PCR for a panel of hypertrophic markers and metabolic genes [16] (Online Supplement Methods).

Liver lipid content was assessed in frozen tissue sections (12 μm), fixed in 4% PFA, rinsed with 60% triethyl phosphate, stained with oil red O (0.5%) and counterstained with haematoxylin.

### 2.5 CardioNet metabolic network reconstruction

In silico simulations were performed using the metabolic network of the cardiomyocyte CardioNet (Online Supplement Methods) [17].

### 2.6 Data analysis and statistics

All data was analysed blind to genotype. Comparison between two groups was by Student's t-test (Gaussian data distribution), Mann–Whitney U test (non-Gaussian data distribution) and between 3 groups by one-way analysis of variance (ANOVA) using Bonferroni's correction for multiple comparisons. Kaplan–Meier survival curves were compared by log-rank test. Pearson's correlation coefficient was used to assess the relationship between the variables. Data are presented as mean ± S.E.M. Statistical analysis was carried out using GraphPad Prism software, v. 5.04. Differences were considered significant when P < 0.05.

## 3 Results

### 3.1 MCD knockout mice exhibit significantly higher early mortality

**Fig. 1:**
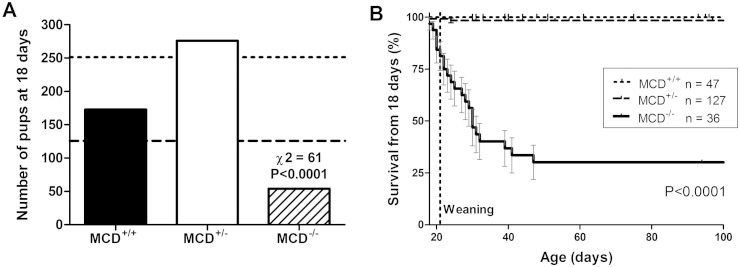
Early mortality results in non-Mendelian genotype ratios at 18 days of age with 53% fewer MCD^−/−^ mice than expected (A). The dashed line represents expected frequency for MCD^−/−^, while the dotted line shows expected frequency for MCD^+/−^. From a total of 503 genotyped off-spring, only 54 MCD^−/−^ mice were identified at 18 days of age. At this point, n = 18 were used for biochemical phenotyping, with the remaining n = 36 contributing to the Kaplan–Meier survival curve (B). Mortality remained high in the peri-weaning period with a further 70% of MCD^−/−^ dying by the age of 50 days.

After establishing the mouse colony using heterozygote pair breeding, non-Mendelian offspring ratios were noted at time of ear-notch sampling for genotype analysis (18 days of age): from 503 pups observed, the frequency at 18 days was MCD^+/−^ 276: MCD^+/+^ 173: MCD^−/−^ 54 (Chi squared = 61; P < 0.0001; Fig. 1A). A further five MCD^−/−^ mice were identified from cadaver remains at between 1 and 16 days of age. Since the expected Mendelian outcome was for 126 MCD^−/−^, this suggests that approximately 53% of MCD^−/−^ pups are unaccounted for, presumably dying either in utero or in post-partum and cannibalised.

Furthermore, mortality remained high throughout the peri-weaning period. Of the 54 MCD^−/−^ alive at 18 days, n = 18 were culled at this time point for biochemical analysis (see below), while the remaining n = 36 were followed into adulthood with 70% dying or euthanized due to illness over the next 4 weeks (P < 0.0001; Fig. 1B).

The majority of these mice were found dead without obvious symptoms despite twice daily observations. A small number were observed to become symptomatic e.g. pilo-erection, laboured breathing, and unresponsive to stimuli (see Table 3 of Online Supplement for details). When anaesthetised with isoflurane to perform an echocardiogram, all mice died within minutes of induction, with contractile function rapidly tapering to zero, consistent with acute decompensated heart failure. Post-mortem examination showed heart-weight ~ 2-fold higher than wild-type (P < 0.004) and confirmed milk in the gastric contents indicating that all mice were suckling normally prior to death.

### 3.2 Altered gross morphology in young MCD knockout mice

**Fig. 2:**
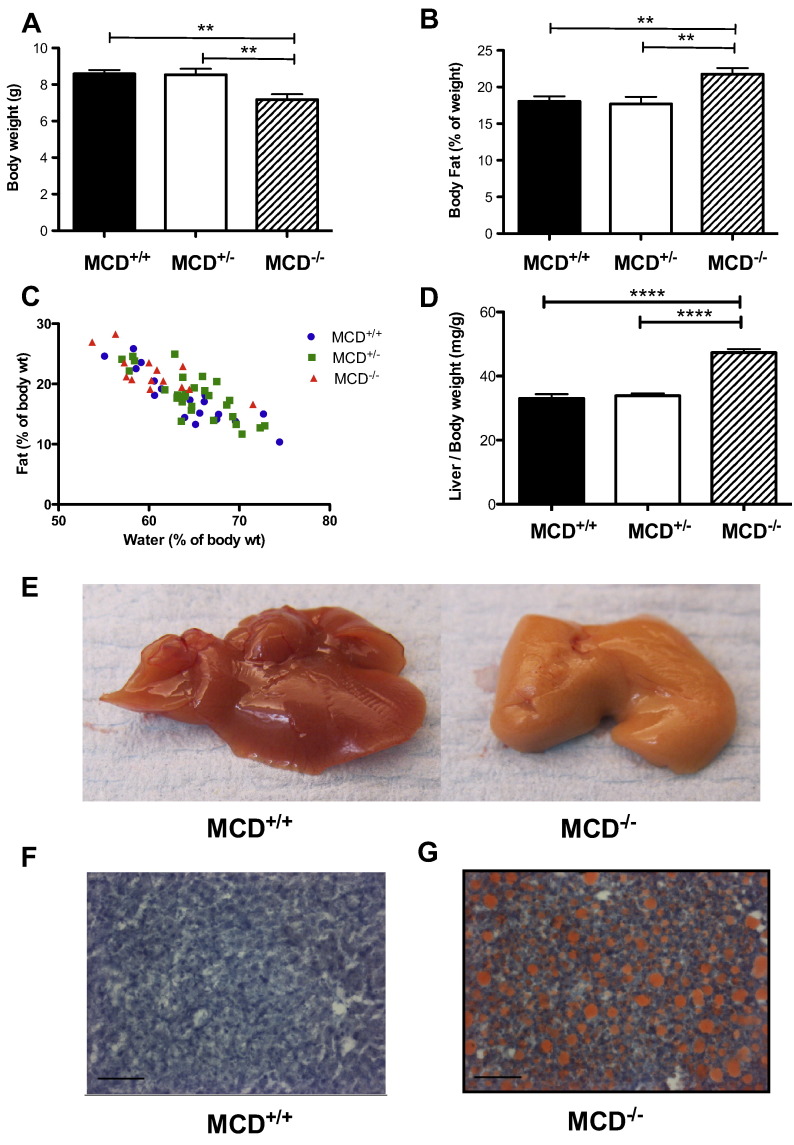
Whole body phenotype of 18 day old MCD^−/−^ mice. (A) Decreased body weight (B) accompanied by increased body fat (C) and decreased total body water % in relation to % body fat measured by in vivo non-invasive magnetic resonance relaxometry (WT Pearson r = − 0.88, p < 0.0001; MCD^+/−^ Pearson r = − 0.7844, p < 0.0001; MCD^−/−^ Pearson r = − 0.7631 p < 0.001) (D). Significantly increased liver weight (E) and “fatty liver” appearance of MCD^−/−^ liver vs MCD^+/+^ (F, G). Representative oil red O stained liver sections revealing lipid deposition (red) in MCD^−/−^ mice that are absent in MCD^+/+^. Images taken at × 20 magnification (marker bar = 0.01 μm). MCD^+/+^ (n = 19), MCD^+/−^ (n = 29), MCD^−/−^ (n = 14).*** denotes P < 0.001, and ** denotes P < 0.01.

MCD^−/−^ mice were systematically studied at 18 days of age since this represents a functional and metabolic snap-shot immediately prior to the period of highest mortality. At 18 days of age, MCD^−/−^ animals were characterized by a significantly reduced body weight (Fig. 2A) and growth retardation [(tibia length (mm) MCD^+/+^: 12.4 ± 0.1 (n = 28); MCD^+/−^ 12.3 ± 0.1 (n = 33); MCD^−/−^ 11.8 ± 0.1 (n = 17); p < 0.004)]. Paradoxically, despite lower body weight, percentage body fat was disproportionately higher in MCD^−/−^ mice versus wild-types (Fig. 2B), whereas total water was in normal proportion to body weight (Fig. 2C).

Livers from MCD^−/−^ mice were heavier in proportion to body weight (Fig. 2D) and were uniformly pale in colour in comparison to wild-type livers (Fig. 2E). Oil red O staining in histological sections (Fig. 2F–G) confirmed the presence of severe hepatic steatosis.

### 3.3 MCD deletion results in pre-weaning cardiac dysfunction

**Fig. 3:**
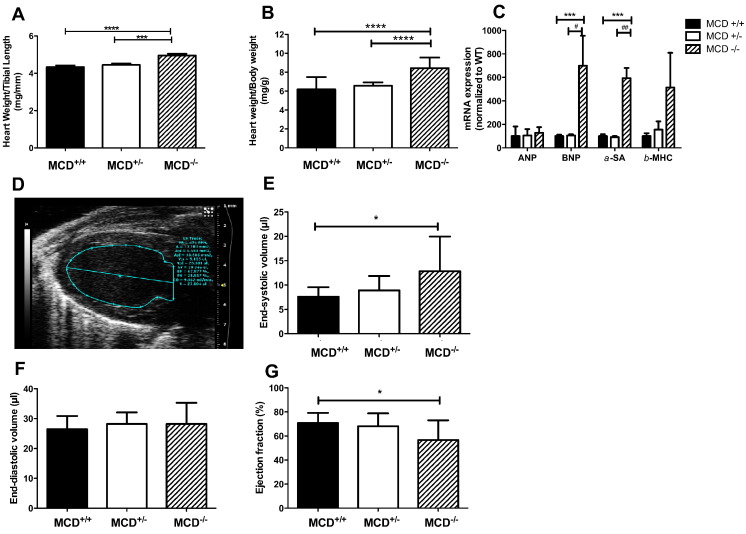
Cardiac phenotype in 18 day old MCD^−/−^ mice. (A, B) Post-mortem hypertrophy indices (MCD^+/+^ n = 27, MCD^+/−^ n = 33, MCD^−/−^ n = 17). (C) Hypertrophy marker mRNA levels. (D) Representative parasternal long-axis echocardiogram from 18 day old MCD^−/−^ mouse. (E-G) Echocardiography analysis of cardiac function [(n = 10, 3male/7female)]. *** denotes P < 0.001, and * denotes P < 0.05 compared to MCD^+/+^. ^##^P < 0.001 versus MCD^+/−^, ^#^P < 0.05 versus MCD^+/−^.

At 18 days of age MCD^−/−^ had LV hypertrophy, as observed from post-mortem weights normalised to body weight or tibia length (Fig. 3A, B).

This was confirmed at the molecular level by elevated mRNA expression of classical hypertrophy markers (Fig. 3C). In vivo echocardiography in 18 day old MCD^−/−^ mice (Fig. 3D) revealed normal LV end-diastolic chamber volume (Fig. 3F), but with increased end-systolic volume (Fig. 3E) resulting in significantly reduced cardiac ejection fraction (Fig. 3G).

Heart rates were comparable and there were no significant differences in Doppler flow indices for mitral valve or pulmonary artery (see Online Supplement Table 4). Simultaneous ECG measurements showed normal interval durations and no arrhythmic events were noted in the 18 day old mice (see Online Supplement Table 5).

### 3.4 Young MCD knockout mice have severely altered metabolic phenotype

**Fig. 4:**
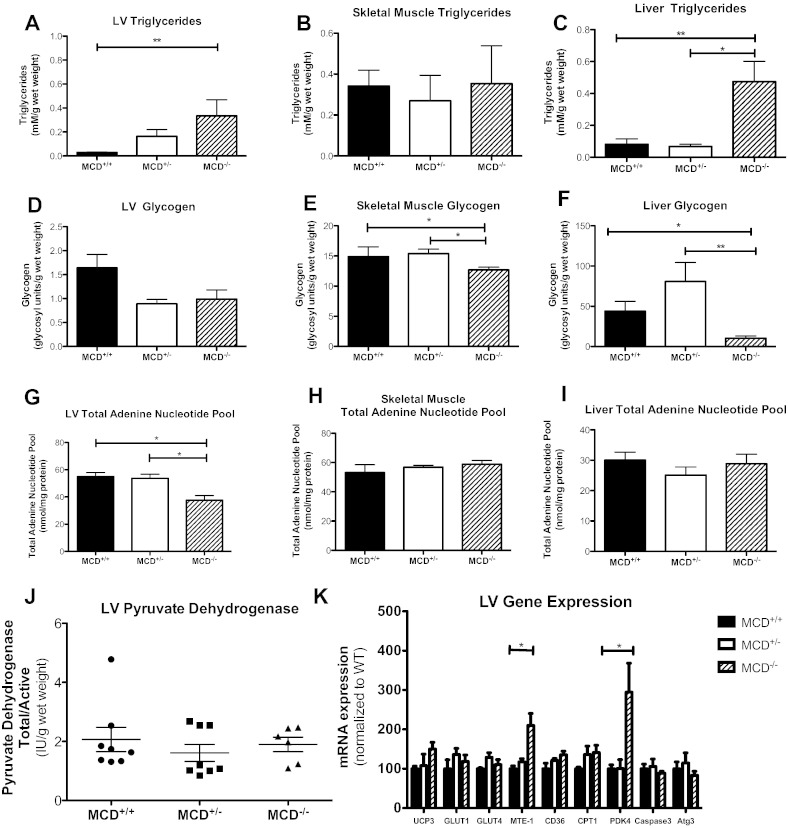
Cardiac metabolic phenotype in MCD^−/−^ mice at 18 days of age. Endogenous energy reserve profile of heart, skeletal muscle (gastrocnemius and soleus) and liver from MCD^−/−^ mice at 18 days of age: (A) LV triglyceride content (all groups n = 4). (B) Skeletal muscle triglyceride content (MCD^+/+^ n = 4, MCD^+/−^ n = 7, MCD^−/−^ n = 7). (C) Liver triglyceride content (MCD^+/+^ n = 5, MCD^+/−^ n = 4, MCD^−/−^ n = 5). (D) LV glycogen content (MCD^+/+^ n = 7, MCD^+/−^ n = 4, MCD^−/−^ n = 4). (E) Skeletal muscle glycogen content (MCD^+/+^ n = 9, MCD^+/−^ n = 4, MCD^−/−^ n = 4). (F) Liver glycogen content (MCD^+/+^ n = 5, MCD^+/−^ n = 4, MCD^−/−^ n = 5). (G) LV total adenine nucleotide (TAN) pool (all groups n = 4). (H) Skeletal muscle total adenine nucleotide pool (all groups n = 4). (I) Liver total adenine nucleotide pool (all groups n = 4). (J) Pyruvate dehydrogenase complex activity [PDH_total_:PDH_active_ ratio: MCD^+/+^ (n = 8), MCD^+/−^ (n = 8), MCD^−/−^ (n = 4)]. (K) Cardiac expression of genes involved in the regulation of fatty acid oxidation (n = 4 per group), apoptosis and autophagy. * denotes P < 0.05 and ** denotes P < 0.01 for MCD^+/+^ or MCD^+/−^ versus MCD^−/−^ group.

As expected, MCD^−/−^ hearts were characterized by elevated malonyl-CoA tissue concentration (C57BL/6J control 2.2 ± 0.3, MCD^+/−^ 5.2 ± 0.3, MCD^−/−^ 4.9 ± 0.7 nmol/mg protein, P < 0.01, n = 3–4). We sought to determine if there were changes in energy storage within heart, skeletal muscle and livers of the MCD^−/−^ mice due to the central role of malonyl CoA in these tissues to regulate mitochondrial fatty acid metabolism in response to changes in cellular fuel availability and energy expenditure [18]. Triglyceride content was 11-fold higher in MCD^−/−^ hearts than in wild-type controls (Fig. 4A). This significant lipid accumulation was accompanied by a trend towards decreased myocardial glycogen content (60% of WT) (Fig. 4D), and a 32% reduction in total adenine nucleotide (TAN) pool (AMP + ADP + ATP) (Fig. 4G), suggesting an energy depleted state in young MCD^−/−^ hearts. LV creatine content was not altered (MCD^+/+^: 70 ± 4, MCD^+/−^: 72 ± 6, MCD^−/−^: 72 ± 3 nmol/g protein; n = 4). Skeletal muscle from MCD^−/−^ animals had significantly depleted glycogen content versus MCD^+/+^ and MCD^+/−^ (Fig. 4E), however, neither triglycerides (Fig. 4B), the TAN pool (Fig. 4H) nor creatine content (MCD^+/+^: 180 ± 13, MCD^+/−^: 172 ± 5, MCD^−/−^: 172 ± 10 nmol/g protein; n = 4) was affected. Similar to LV tissue, chronic MCD deficiency had profound effects on the liver metabolic phenotype, with triglyceride content 5.9-fold higher (Fig. 4C) and glycogen stores virtually depleted (Fig. 4F). Liver TAN pool was not affected (Fig. 4I).

MCD^−/−^ hearts were also characterized by decreased citrate synthase activity (MCD^+/+^ 274 ± 43; MCD^+/−^ 381 ± 24; MCD^−/−^ 156 ± 26 IU/g wet weight, n = 4, P < 0.05), which may reflect changes in mitochondrial volume. Furthermore, there was no adaptive increase in pyruvate dehydrogenase activity (Fig. 4J), suggesting that flux through glucose oxidation was unchanged in the setting of chronically and severely inhibited fatty acid oxidation.

In MCD deficient hearts, there was an increase in mRNA levels of mitochondrial thioesterase-1 (MTE-1) and PDK4 without changes in expression for the other genes examined: uncoupling protein 3 (UCP 3), glucose transporters (GLUT1, GLUT4), fatty acid transporter (CD36), and mitochondrial fatty acid transporter carnitine palmitoyltransferase 1 (CPT1) (Fig. 4K). There were no changes in the expression of the markers of apoptosis (caspase 3) and autophagy (Atg3) [19] (Fig. 4K).

**Fig. 5:**
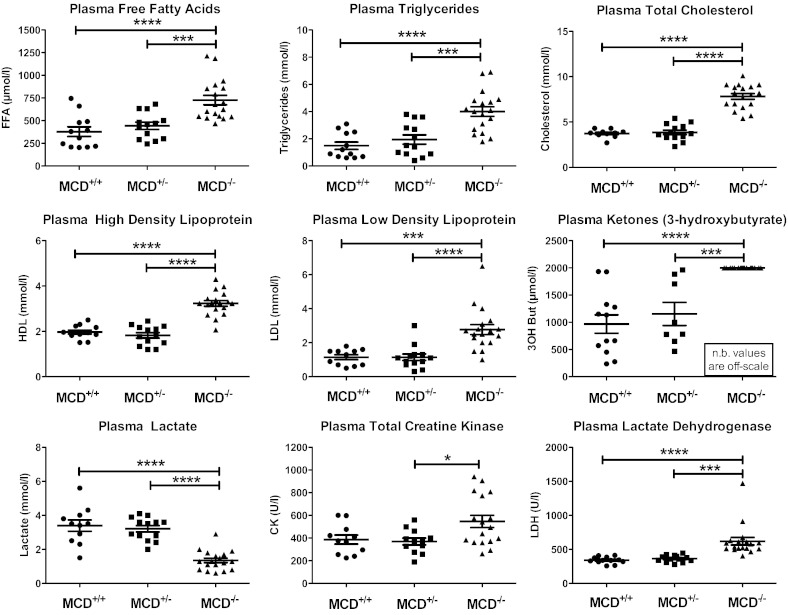
Plasma metabolite concentrations in MCD^−/−^ mice at 18 days of age. (MCD^+/+^ n = 12, MCD^+/−^ n = 13, MCD^−/−^ n = 18) FFA — free fatty acids, LDL — low density lipoprotein, HDL — high density lipoprotein, and LDH — lactate dehydrogenase. *** denotes P < 0.001 and * denotes P < 0.05 compared to MCD^+/+^ or MCD^+/−^.

Plasma collected from 18 day old MCD^−/−^ mice was milky in appearance due to very high lipid composition: free fatty acids, triglycerides, total high density lipoprotein and low density lipoprotein cholesterol were all ~ 2-fold above wildtype levels (Fig. 5). Circulating ketone bodies (3-hydroxybutyrate) were above the upper limit of detection for this assay and plasma lactate was low in KO, suggesting increased utilization as an alternative fuel (Fig. 5). Plasma lactate dehydrogenase and total creatine kinase levels were both elevated in MCD^−/−^ (Fig. 5), indicating cellular damage and (combined with elevated ketone bodies) a grossly catabolic starvation state.

### 3.5 Early cardiac dysfunction phenotype resolves with age

**Fig. 6:**
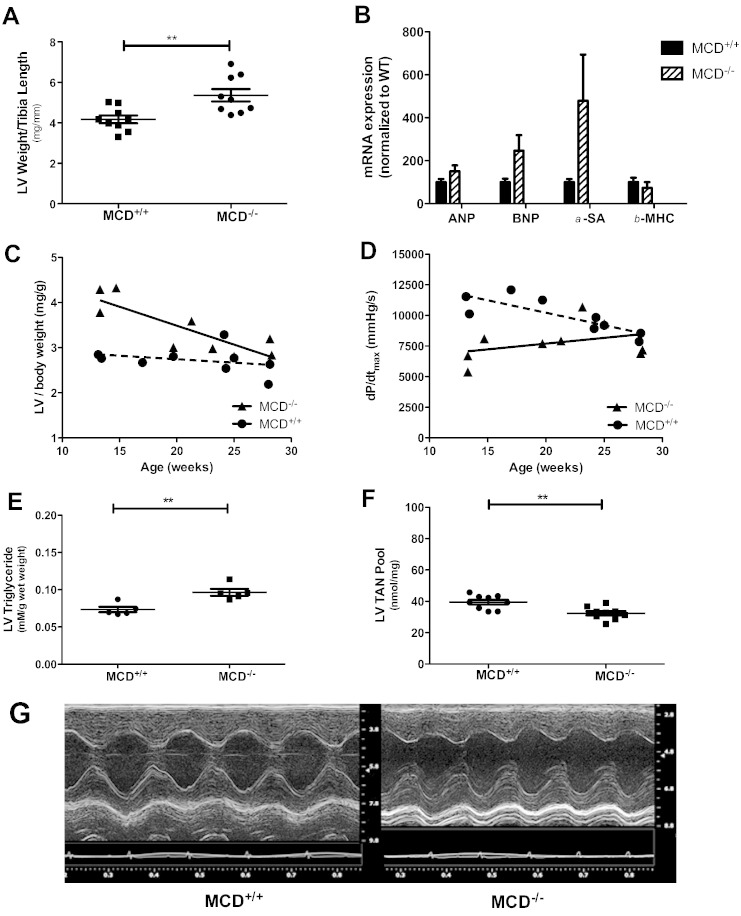
Cardiac phenotype in adult MCD^−/−^ mice. (A) Assessment of cardiac hypertrophy at morphological and molecular level; (B) mRNA levels of cardiac hypertrophic markers (n = 9 per group). (C) Relationship between LV weight (MCD^−/−^ r = − 0.83, P = 0.005; MCD^+/+^ r = 0.31, P = 0.42) and (D) contractility (MCD^−/−^ r = 0.36, P = 0.34; MCD^+/+^ r = – 0.82, P = 0.007) versus age. (E) Metabolic (triglyceride) and (F) energetic (total adenine nucleotide pool) profile of MCD surviving adult mice (n = 5–9 per group). (G) Representative M-mode ultrasound images obtained from the parasternal short-axis view in MCD adult mice.

The nine MCD^−/−^ mice that survived > 50 days of age were used for morphological, in vivo echocardiography and hemodynamic assessment and compared to sex and age matched WT controls. Despite the significant differences in animal gross body morphology observed at the young age, body weights of adult MCD^−/−^ mice were comparable to wild-type (Table 1). LV hypertrophy was still detectable by post-mortem heart weight (Table 1), and when normalised to tibia length (Fig. 6A) and body weight (Table 1). However, hypertrophy was no longer evident at the molecular level, with mRNA of key hypertrophic markers no longer significantly elevated above wild-type (Fig. 6B).

**Table 1:** Morphometric and hemodynamic parameters of MCD^−/−^ survivor mice.

	Adult MCD^+/+^ (n = 6 F + 3 M)	Adult MCD^−/−^ (n = 6 F + 3 M)	P value
Age (weeks)	21 ± 2	21 ± 2	0.81
Organ weights			
Body weight (g)	28.2 ± 1.7	29.5 ± 2.7	0.69
LV/body weight (mg/g)	2.72 ± 0.10	3.42 ± 0.20	0.006
RV/body weight (mg/g)	0.79 ± 0.10	0.99 ± 0.06	0.007
Lung/body weight (mg/g)	4.8 ± 0.8	4.8 ± 1.2	0.98
Liver weight/body weight (mg/g)	42.9 ± 1.4	41.8 ± 1.5	0.63
Echocardiography			
End diastolic area (mm^2^)	9.3 ± 0.4	10.6 ± 0.6	0.10
End systolic area (mm^2^)	3.2 ± 0.5	4.1 ± 0.8	0.39
Fractional area change (%)	66 ± 4	63 ± 5	0.65
Myocardial CSA (mm^2^)	10.8 ± 0.5	13.1 ± 0.7	0.02
PAAT (ms)	18.1 ± 1.3	17.7 ± 1.4	0.86
Hemodynamics			
Heart rate (bpm)	505 ± 16	493 ± 20	0.66
LV systolic pressure (mm Hg)	98 ± 2	96 ± 2	0.50
LV end-diastolic pressure (mm Hg)	6 ± 0.7	8 ± 0.9	0.12
dP/dt_max_ (mm Hg/s)	9929 ± 483	7769 ± 514	0.007
dP/dt_min_ (mm Hg/s)	− 9270 ± 589	− 7105 ± 995	0.08
Tau (ms)	6.9 ± 0.4	9.1 ± 0.8	0.04
Stimulated dP/dt_max_ (mm Hg/s)	13895 ± 957	10505 ± 1045	0.03

LV — left ventricular, RV — right ventricular, CSA — cross sectional area, and PAAT — pulmonary artery acceleration time. All values are mean ± SEM. Comparisons were made by Student's t-test. Stimulated dP/dt denotes value obtained during maximal stimulation with dobutamine 16 ng/g BWt/min.

When indexed LV weight was plotted against age there was a clear convergence between knockout and wild-type with time, indicating a gradual normalization of LV hypertrophy with increasing age (Fig. 6C, D).

MCD^−/−^ adult mice had normal LV chamber areas, LV pressures and heart rate (Table 1), but the rate of pressure development and relaxation was impaired (dP/dt_max_ and Tau) (Table 1). ECG interval parameters were normal with the exception of a slightly prolonged QRS interval in MCD^−/−^, which most likely reflects residual LV hypertrophy in these animals. An irregular R–R interval was noted in n = 1 MCD^+/+^ and n = 1 MCD^−/−^ during echocardiography, but was not observed during subsequent LV cannulation (Online Supplement Table 6).

Again, differences between MCD^−/−^ and MCD^+/+^ converged with age, suggesting a gradual attenuation of the early cardiac dysfunction. In support of this, there was a marked improvement in metabolic phenotype in older mice: 11.5 fold elevation in the heart TAG content observed in the pre-weaning MCD knockout animals was reduced to 1.3 fold difference in the young adult hearts (Fig. 6E), liver weight to body weight was comparable to wild-types with normal colouration (Table 1), and the 32% reduction in cardiac TAN pool became an 18% difference (Fig. 6F).

### 3.6 In silico modelling of MCD^−/−^ metabolism with high and low fat diets

Our experimental data suggests normalization of cardiac and metabolic phenotypes as MCD^−/−^ reach adulthood. However, the very high levels of early mortality severely limited our ability to perform additional experiments in adult mice and we therefore estimated metabolic flux rates by using the metabolic network reconstruction of the human cardiomyocyte CardioNet [17]. In particular, we sought to determine whether the major change in dietary composition that occurs at weaning could contribute to the observed phenotypic resolution. Simulations of MCD deletion were run under conditions of high dietary fat to mirror the high-fat composition of maternal milk and re-run under low-fat availability to reflect standard mouse chow consumed post-weaning (Supplementary Table 2).

**Fig. 7:**
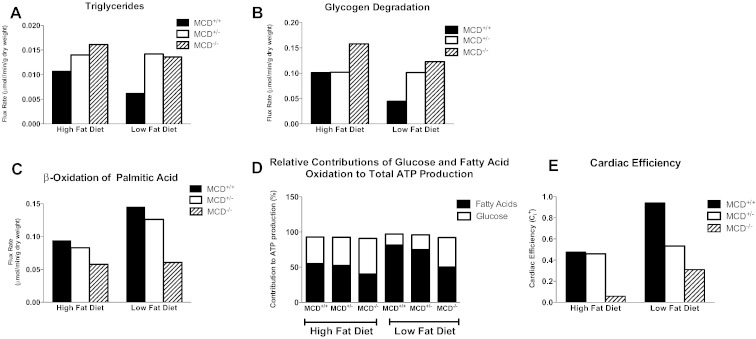
CardioNet in silico mathematical simulation results of MCD^+/+^, MCD^+/−^, and MCD^−/−^ mice under high-fat and low-fat dietary conditions: (A) Endogenous myocardial triglyceride synthesis. Flux rates of triglyceride synthesis in μmol/min/g dry weight. (B) Glycogen degradation. Flux rates are given in μmol/min/g dry weight. (C) Rate of palmitic acid β oxidation. Flux rate in μmol/min/g dry weight. (D) Relative percentage contribution of fatty acid and glucose oxidation rates to total ATP production. (E) Impact of dietary fat composition on cardiac efficiency.

High-fat simulations re-capitulated the experimental findings showing overt triacylglyceride accumulation with MCD deficiency (Fig. 7A). Furthermore, MCD insufficiency increased the flux rate for glycogen degradation which agrees with depletion of glycogen content observed experimentally (Fig. 7B). Despite the increased availability of fatty acids with high-fat diet, the contribution of palmitic acid to β-oxidation in MCD deficiency was lower in MCD^−/−^ (Fig. 7C) and the relative contribution of glucose to acetyl-CoA production was predicted to be higher (Fig. 7D). Of particular note, metabolic changes caused by the combination of MCD deficiency and high-fat diet translated into a greatly reduced cardiac efficiency, which dramatically improved when switched to a low-fat diet (Fig. 7E).

MCD^+/−^ in silico modelling data revealed that in early life, under high fat diet conditions, heterozygous mice have a metabolic profile comparable to MCD^+/+^ in terms of fatty acid oxidation (Fig. 7C), glucose vs. fatty acid contribution (Fig. 7D) and cardiac efficiency (Fig. 7E) thus recapitulating our experimental findings. However, long term they may develop an intermediate phenotype characterized by mild fatty acid metabolism disorder based on the prediction of increased TAG synthesis and glycogen breakdown.

## 4 Discussion

The major finding of this study is that cardiac dysfunction was universally observed in all MCD knockout mice, resulting in high peri-natal and peri-weaning mortality. We believe that death was due to acute decompensated heart failure since animals that were found symptomatic had laboured breathing and pump failure, followed by death, which was directly observed using ultrasound in the absence of arrhythmia on the ECG. However, we cannot rule out a whole body metabolic component. The fact that cardiac function was only mildly impaired at 18 days of age suggests that decompensation can occur very rapidly in the mouse, and this is consistent with our own observations using surgical models of heart failure.

Our results further suggest that cardiac metabolic impairment precedes dysfunction as the intrinsic feature of MCD deficiency during infancy, and that variable presentation in patients likely reflects dietary and nutritional factors. These conclusions based on our biochemical experimental data were reiterated by in silico metabolic modelling.

Our data presents a compelling narrative for the evolution of a cardiac dysfunction phenotype in MCD deficiency. In the post-natal period there is increased dependence on fatty acid oxidation concomitant with the sudden increase in cardiac work and abundance of fatty acids in the maternal milk supply [20]. Suckling represents an exclusively high-fat diet (milk from C57BL/6 dams is 42% fat [21]), that MCD knockout mice are unable to fully utilize. This resulted in grossly abnormal lipid accumulation as evidenced by increased body fat composition, dramatically elevated LV and liver triglyceride levels, and hyperlipidemic serum. Intra-cardiac accumulation of triglyceride in MCD^−/−^ hearts may have direct toxic effects on cardiomyocytes, as previously described in analogous models of fatty acid accumulation. For example, carnitine-deficient mice [22], high-fat feeding and streptozotocin induced diabetes [23], where elevated LV triglyceride levels were linked to poor survival, cell death and cardiac dysfunction [24]. In parallel, we observe a reduced body weight, depleted tissue glycogen, and reduced serum lactate levels, which may be used as an alternative carbohydrate source [25].

These changes are all indicative of a catabolic state and starvation phenotype caused by the depletion of alternative substrates due to inability to utilize fatty acids. Protein catabolism is suggested by a massive increase in ketone bodies, which may exacerbate the phenotype by affecting cellular fatty acid uptake [26], while high LDH and CK activities reflect muscle damage/breakdown. The failure to compensate via an increase in PDH-mediated glucose oxidation is likely to be critical in the evolution of energy depletion. In this regard, up-regulation of PDK4 mRNA is a particular maladaptation since it is a key inhibitor of PDH [27]. Reduced ATP supply could also be due to lower mitochondrial volume as implied by reduced activity of citrate synthase.

The outcome is reduced adenine nucleotides in the heart, a state that is usually only observed following acute ischemia or in chronic heart failure, where levels correlate with severity of cardiac dysfunction [28].

The additional challenge of weaning, when pups are abruptly removed from the dam, results in reduced calorific intake until pups learn to take solid food. It is likely that this challenge is sufficient to kill many that are already energy starved. Weaning also represents an abrupt change in dietary composition (42% fat content in milk to 4% in chow). Surviving knockout mice appear to re-energise the heart on a standard low-fat diet where 62% of the calorific value comes from carbohydrate, resulting in gradual resolution of cardiac dysfunction and improving tissue lipid accumulation.

### 4.1 In silico modelling

The abnormally high mortality in young MCD^−/−^ mice meant that there was very little adult tissue for metabolic phenotyping. For example, we would have liked to measure exogenous substrate oxidation (e.g. palmitate), but this typically requires n = 15 per group [6]. Since we only obtained nine adult MCD^−/−^ from 503 offspring, breeding sufficient mice for such experiments would be prohibitive. Thus to test the hypothesis that changes in dietary substrate availability account for resolution of the MCD^−/−^ phenotype, we used an established computer model of cardiomyocyte metabolism [17] to simulate the switch from maternal milk to standard laboratory chow. This approach has several inherent limitations.

For example, no account was taken of developmental changes in activity or expression of metabolic enzymes between young and adult hearts, although this also represents an advantage, since it allowed us to ask whether the change in substrate availability is, in itself, sufficient to improve the cardiac metabolic phenotype. The model takes no account of potential differences in neuroendocrine stimulation and there may be species differences since the model is, by necessity, chimeric. Simulated high-fat diet was based on published values for mouse milk taking into account plasma substrates measured in 18 day old mice. Equivalent data was not available for adult mice and therefore low-fat diet was based solely on the composition of chow with no account of potential differences in bioavailability.

Despite these shortcomings, the use of the model is validated by recapitulation of our key experimental findings, e.g. triacylglyceride accumulation and glycogen depletion under high-fat conditions, and is in agreement with the previously published observations in MCD^−/−^ mice [29].

Moreover, the agreement between intermediate metabolic phenotype of MCD^+/−^ simulations with the actual experimental findings further validates the use of in silico modelling.

The modelling data indicates a move towards normalization of the MCD^−/−^ metabolic phenotype associated with an improvement in cardiac efficiency when switched from milk to a standard chow diet. This supports the hypothesis that dietary substrate availability is a major determinant of the MCD deficiency phenotype.

### 4.2 Comparison with published MCD knockout phenotype

The level of early mortality observed here was not originally reported in the MCD colony we obtained these mice from, despite having a similar pathogen-free status and using identical chow. Nevertheless, MCD gene deletion results in ~ 30% pre-weaning mortality in the originating MCD^−/−^ colony (personal communication Prof Lopaschuk, 2014). One key difference between colonies is in breeding strategy, where we used Het pairs to obtain a mix of genotypes, while Dyck et al. used KO pair breeding. It is possible that MCD gene deletion alters the fat composition of milk leading to MCD^−/−^ dams providing a lower fat load to suckling pups than the corresponding MCD^+/−^ dams. Nor can we exclude the possibility of differences in placental metabolite supply in utero potentially predisposing MCD^−/−^ to worse survival outcome. In addition, MCD^−/−^ mice are small and under-developed, therefore mice in litters containing only knockouts do not have to compete with stronger siblings for maternal milk supply. We planned to test this experimentally, but after > 18 months of breeding have failed to obtain the necessary MCD^−/−^ adult breeding pairs.

Another difference between the originating MCD colony and the Oxford colony used in this study is the provenance of the C57BL/6 sub-strain used for backcrossing. The Oxford colony is congenic to C56BL/6J supplied by Harlan UK, whereas the Alberta colony is on a JAX™ C57BL/6 background from Charles River. The latter is known to have a mutation in the Nnt (nicotinamide nucleotide transhydrogenase) gene, which is unaffected in the Harlan UK sub-strain [30]. Nnt encodes a mitochondrial protein that catalyses the production of NADPH and mice with the Nnt mutation develop asymptomatic metabolic syndrome characterized by glucose intolerance and obesity-independent reduced insulin secretion [31], [32]. This genetic divergence may be one of the factors contributing to the difference in survival between these two colonies and it is likely that there will be other, as yet unidentified, incidences of genetic drift between these C57BL/6J sub-strains.

Other environmental factors may also be important in deciding survival, e.g. litter size and husbandry practice at time of weaning. Nevertheless, by the time mice were fully adult, cardiac function was within the normal range in agreement with the published literature [5], [6], [7].

### 4.3 MCD heterozygote phenotype and malonyl-CoA levels

It is notable that malonyl-CoA levels in MCD^+/−^ mice were similar to MCD^−/−^, yet MCD^+/−^ had less triglyceride accumulation in the heart, suggesting a limited ability to utilize fatty acids remained, and was sufficient to maintain TAN pool and normal cardiac function. Therefore, the inhibition of CPT-1 by malonyl-CoA is not the sole driver of the MCD^−/−^ phenotype and the loss of MCD protein itself is also relevant. This suggests that alternative roles exist for MCD control of lipid metabolism, potentially related to the high abundance of malonyl-CoA and MCD located in cardiac peroxisomes [1], [33].

### 4.4 Implication for human MCD and lipid oxidation deficiency syndromes

Our study demonstrates for the first time that in early life cardiac dysfunction is a consistent finding directly related to MCD deficiency, and that variable penetrance of the phenotype in patients likely reflects environmental factors linked to alternative substrate availability. Furthermore, our study suggests that heart screening should be a routine investigation in these patients, with extra monitoring focused on times of enforced fasting, concurrent illness or major dietary change.

The structural, functional and metabolic changes observed in MCD^−/−^ mice at the post-weaning stage are characteristic of cardiomyopathies caused by mitochondrial fatty acid metabolism defects which typically manifest early in life and are associated with cardiac remodelling, conduction defects and sudden onset of heart failure [20], [34], [35]. For example, the observed metabolic phenotype has similarities to CPT-1 deficiency described in Inuit patients on traditional diets rich in fat and low in carbohydrate that result in high infant mortality [36].

Currently, there are no established dietary and therapeutic managements for MCD deficiency disorder [12], [37], we therefore propose that MCD^−/−^ mice would make a good model for future experimental studies. Furthermore, heterozygous MCD^+/−^ animal would be an interesting model of mild MCD deficiency for future work.

### 4.5 Implications for therapeutic modulation of MCD

Pharmacological MCD inhibitors are under development e.g. for treatment of cardiac ischaemia [6] and obesity [38]. Our study suggests that caution is required with therapeutic inhibition of fatty acid oxidation, a view further supported by a recent study in CPT-1 heterozygous knockout mice subjected to aortic banding, which displayed mitochondrial abnormalities, lipid accumulation, and had greater functional impairment and mortality compared to controls [39]. Whether there are circumstances in which cardiac dysfunction may be induced by MCD inhibition in an adult population merits further study.

## 5 Conclusion

This is the first study to demonstrate that energy starvation and cardiac insufficiency are intrinsic features of chronic MCD deficiency during the suckling and peri-weaning period. It suggests that phenotype severity likely depends on the composition of food intake and that high-fat, low carbohydrate diets or periods of enforced fasting are particular risk factors. The MCD^−/−^ mouse could represent a suitable model to test therapeutic dietary modulation for patients with inborn errors in fatty acid metabolism.

## Disclosures

GDL is a major shareholder in Metabolic Modulators Research Ltd, which is developing MCD inhibitors for treatment of heart disease in adults.
