# Supplementary material for: Cardiac dysfunction and peri-weaning mortality in malonyl-coenzyme A decarboxylase (MCD) knockout mice as a consequence of restricting substrate plasticity
Source: J Mol Cell Cardiol. 2014 Oct;75:76–87. doi: 10.1016/j.yjmcc.2014.07.008 (PMC4169183; doi:10.1016/j.yjmcc.2014.07.008)
Supplement: Supplementary file 1 — Supplementary material. [file mmc1.pdf]

Online Supplementary Material

Supplementary Methods

Pyruvate Dehydrogenase Assay

The active fraction of pyruvate dehydrogenase (PDH_a) was extracted from powdered myocardial tissue using buffer containing (mmol/L) HEPES 25, ADP 1, EDTA 1, dichloroacetate 3, KH_2PO_4 25, dithiothreitol (DTT) 1, leupeptin 0.05, KF 25 and 1%(v/v) triton X-100 (pH 7.0) to inhibit PDH phosphatase and PDH kinase as described previously [1]. Tissue for total PDH activity (PDH_t) analysis was extracted using buffer containing (mmol/L): HEPES 75; dichloroacetate 5; $MgCl_2$ 5; ADP 1; dithiothreitol (DTT) 1; leupeptin 0.05; and 1% Triton X-100, pH 7.0. Samples were freeze-thawed 3 x 30 sec and resulting mixture was centrifuged at 13 000rpm, 4°C. PDH activity was measured spectrophotometrically at 340 nm. Reaction mixture containing (mmol/L): HEPES 50; $MgCl_2$ 1; EGTA 0.08; DTT 1; NAD 1.67; Co-enzyme A 0.2; thiamine pyrophosphate 0.2; lactate 16.7; rotenone 4µM; lactate dehydrogenase 2U- was incubated at 30°C for 5 minutes prior to the addition of tissue sample and the rate of NADH production followed over 1 min.

Tissue Glycogen and Triglycerides Assessment

In brief, glycogen was extracted by alkali digestion by dissolving approximately 40 mg tissue in 30% (w/v) potassium hydroxide and at 100°C for 30 min. Subsequently, 2% (w/v) anhydrous sodium sulphate and sufficient 100% (v/v) ethanol to give a final concentration of 75% were added. Samples were centrifuged at 27 000g for 10 min at 4°C and the pellet was rinsed with 80% ethanol. The resultant glycogen pellet was dried at 37°C for 30 min and digested in 0.5M amylo-α-1,4-α-1,6-glucosidase, 1M sodium acetate buffer pH 5.0 for 1 hour at 37°C. Glucose concentration of resulting solution was determined spectrophotometrically utilizing glucose oxidase and peroxidase. Sample was incubated with glucose assay reagent (0.1 mmol/L NaH_2PO_4 buffer, pH 7.0, 0.8U peroxidase/ml, 10U glucose oxidase/ml, 0.4mmol/L 4-aminoantipyrine, 10mmol/L 4-hydroxybenzene sulphonic acid) for 30 min at 30°C.

Absorbance was recorded at 510 nm. A standard curve covering 1-5 mmol/L was used for the determining the sample concentrations.

In a separate series of experiments, tissue lipids were extracted from KO, WT and Het mouse tissue samples (LV, skeletal muscle, liver). Powdered tissue was homogenised in ice-cold chloroform-methanol-water mixture (2:1:0.8) and centrifuged for 15 min (13 500 rpm, 4°C). The upper aqueous layer was decanted, the lower chloroform layer dried under 100% gaseous N_2 and re-suspended in 2-propanol. Triglyceride content was assayed using a Sigma-Aldrich kit TR0100 (Sigma-Aldrich, UK).

Real-time quantitative Reverse Transcriptase-Polymerase Chain Reaction

Total RNA (1ng) was used as input in one-step RT and amplification reactions using the Qiagen Quantitect SYBR Green RT-PCR kit (Qiagen, UK) on the Rotor-Gene system (Corbett Research Ltd, Qiagen, UK). The oligonucleotide sequences are listed in Table 1. Either the sense or the antisense oligonucleotide per pair, were designed to span intron-exon boundaries on the cDNA sequence. For data analysis, the double-standard curve method was employed, in which standard curves spanning five log dilutions of heart RNA were constructed for both the reference and the genes of interest. For quantification, the relative quantities of the above genes were normalized against the reference gene 36B4.

CardioNet metabolic network reconstruction

Flux distributions were predicted for different nutritional supplies while making the assumption that in an environment with restricted access to resources cardiac metabolism is following the concept of optimality. Optimal solutions were calculated for a limited set of 10 nutrients while demanding a complex metabolic target function reflecting important cellular functions of the cardiomyocyte as previously described [2].

MCD knockout was simulated by restricting the corresponding network reaction to carry a zero flux. Constraints were applied to the exchange of substrates to reflect the diet depending variability of available nutrients, such as saturated and unsaturated, medium and long-chain fatty acids, glucose, ketone bodies, lactate and pyruvate.

The dietary composition for fatty acids, glucose and ketone bodies were added according to experimentally obtained plasma concentrations for high-fat diet (Figure 5). As the plasma metabolite concentrations were not available under low-fat diet conditions, substrate availability were constrained according to the dietary composition of normal laboratory mouse chow fed to adult MCD animals (Table 2 online supplement). Flux balance analysis was used to simulate MCD deficiency using a PERL based algorithm in conjunction with the solver CPLEX. Cardiac efficiency was calculated based on (i) the oxygen demand and (ii) endogenous glucose demand and (iii) total substrate uptake as previously described [2]. Flux distributions were analyzed in R statistics and Cytoscape [3].

Supplementary Tables

Table 1 Online Supplement. Oligonucleotide primers for metabolic gene mRNA level assessment.

Gene Symbol	Sense	Antisense	Accession No
Glut1	**5’CTTGTGGCCTCTGCTGCT3’**	**5’GCTTCTTCAGCACACTCTTGG3’**	NM_011400.3
Glut4	**5’GGTTGCCCAGGTGCTGGG3’**	**5’GGCAGGCCCCTCCAGG3’**	NM_009204.2
Pdk4	**5’GGGGGCGGCAAGAGCTGCCCG3’**	**5’GGGGCTCTGGATATACCAGCTCTTC3’**	NM_013743.2
Ucp3	**5’ GGGGGCGGACCACTCCAGCG3’**	**5’GGGGGGCTTGAAATCGGACC3’**	NM_009464.3
Cpt1β	**5’GGAAAGGTATGGCCACTT3’**	**5’CCCGTGGTAGGAGAGCAG3’**	NM_009948.2
36B4	**5’AGATTCGGGATATGCTGTTGG3’**	**5’TCGGGTCCTAGACCAGTGTTC3’**	NM_007475.5
MTE-1	**5’GGGGCCTGCCCCCAGAACCT3’**	**5’GGGGGCCAGAGCCATCACGG3’**	NM_134188.3
CD36	**5’GGGGGTGGGCTCATTGCTGGAG3’**	**5’GGGGAGGACAACTTCCCTTTTG3’**	NM_001159555.1
Caspase-3	**5'GGACTGTGGCATTGAGACAG3'**	**5'CGACCCGTCCTTTGAATTTC3'**	NM_001284409
Atg3	**5'TGCGACAGTCTCTCCGTGC3'**	**5'GGCCACTTCCAGAGCCTTTC3'**	NM_026402

Table 2 Online Supplement. Applied dietary composition for CardioNet mathematical simulations

	High-fat diet MCD $^{+/+}$	High-fat diet MCD $^{+/-}$	High-fat diet MCD $^{-/-}$	Low-fat diet MCD $^{+/+}$	Low-fat diet MCD $^{+/-}$	Low-fat diet MCD $^{-/-}$
**Fatty Acids**						
**total**	44.1809%	44.1809%	66.0969%	4.0000%	4.0000%	4.0000%
Palmitate	7.0689%	7.0689%	10.5755%	0.4356%	0.4356%	0.4356%
Stearate	7.0438%	7.0438%	10.5755%	0.4356%	0.4356%	0.4356%
Linoleate	19.3706%	19.3706%	29.0826%	2.2957%	2.2957%	2.2957%
Oleate	7.9243%	7.9243%	11.8974%	0.9248%	0.9248%	0.9248%
Docosahexaenoic acid	1.3254%	1.3254%	1.9829%	0.0015%	0.0015%	0.0015%
Eicosapentaenoic acid	1.3254%	1.3254%	1.9829%	0.0015%	0.0015%	0.0015%
**Glucose**	41.6887%	41.6887%	13.2594%	76.0000%	76.0000%	76.0000%
**3-Hydroxybutyrate**	6.5652%	6.5652%	9.8218%	0.0000%	0.0000%	0.0000%

Results

Table 3 Online Supplement. Fate of MCD$^{-/-}$ mice discovered to be ill during twice daily observations

ID, Sex & Age	Symptoms	Comments
#9707 Male Found ill at 25 days	Hunched; deep slow respiration; slow to respond to stimuli.	Attempted Echo examination, but died within minutes of anaesthesia. Heart grossly dilated and scarcely beating. Liver had nutmeg appearance.
#9724 Male Found ill at 28 days	Hunched; deep slow respiration; slow to respond to stimuli.	Attempted Echo, but died within minutes of anaesthesia. Heart grossly dilated and scarcely beating.
#236 male Found ill at 30 days	Weight loss; hunched; piloerection; labored respiration.	Attempted Echo, but died within minutes of anaesthesia (no images obtained).
#361 Female Found ill at 20 days	Hunched; deep slow respiration; slow to respond to stimuli.	Immediately euthanized.
#597 Male Found ill at 24 days	Deep slow respiration; slow to respond to stimuli.	Attempted Echo, but died within minutes of anaesthesia (no images obtained).<br>Liver had nutmeg appearance.

Table 4 Online Supplement Echocardiographic parameters in 18 day old anaesthetised MCD mice

	**MCD**$^{+/+}$ (n=10)	**MCD**$^{+/-}$ (n=10)	**MCD**$^{-/-}$ (n=10)
Left ventricular parameters			
Heart rate (bpm)	464 ± 14	455 ± 11	426 ± 19
End-diastolic volume (µl)	26 ± 1	28 ± 1	28 ± 2
End-systolic volume (µl)	8 ± 1	9 ± 1	13 ± 2*
Stroke volume (µl)	19 ± 1	19 ± 1	15 ± 1
Cardiac output (ml/min)	8.1 ± 0.7	8.2 ± 0.7	6.3 ± 0.7
Ejection fraction (%)	71 ± 3	68 ± 3	57 ± 5*
Wall thickness diastole (mm)	0.68 ± 0.03	0.67 ± 0.02	0.73 ± 0.02
Wall thickness systole (mm)	1.09 ± 0.04	1.03 ± 0.04	1.02 ± 0.05
Pulmonary artery Doppler			
PAT – acceleration time (ms)	17 ± 1	17 ± 0.4	18 ± 1
PET – ejection time (ms)	65 ± 1	68 ± 1	65 ± 2
PAT/PET	0.26 ± 0.01	0.25 ± 0.01	0.28 ± 0.02
Transmitral Doppler			
E wave (mm/s)	565 ± 35	562 ± 36	510 ± 37
A wave (mm/s)	169 ± 15	178 ± 17	138 ± 9
E/A	3.5 ± 0.3	3.3 ± 0.2	3.8 ± 0.3

Data is mean ± SEM. CSA is cross-sectional area.* denotes $P < 0.05$ between MCD$^{+/+}$ and MCD$^{-/-}$ by one-way ANOVA with Bonferroni’s correction for multiple comparisons.

Table 5 Online Supplement ECG parameters in 18 day old anaesthetised MCD mice

	**MCD**$^{+/+}$ (n=10)	**MCD**$^{+/-}$ (n=10)	**MCD**$^{-/-}$ (n=9)
RR interval (ms)	128 ± 2	140 ± 3	138 ± 8
PR interval (ms)	39 ± 1	39 ± 1	41 ± 1
P duration (ms)	9.1 ± 0.3	9.1 ± 0.4	8.6 ± 0.2
QRS interval (ms)	8.5 ± 0.4	8.5 ± 0.4	8.8 ± 0.5
QT interval (ms)	16.1 ± 1.0	15.8 ± 0.4	17.6 ± 1.1
QTc interval (ms)	45 ± 3	42 ± 1.4	48 ± 3

Data is mean ± SEM. There are no significant differences for any parameter using one-way ANOVA.

Table 6 Online Supplement ECG parameters in anaesthetised MCD mice that survived into adulthood

	**MCD**$^{+/+}$ (n=9)	**MCD**$^{-/-}$ (n=9)
RR interval (ms)	121 ± 3	121 ± 2
PR interval (ms)	44 ± 1	44 ± 2
P duration (ms)	8.8 ± 0.3	8.7 ± 0.3
QRS interval (ms)	9.5 ± 0.2	10.5 ± 0.2*
QT interval (ms)	17.1 ± 0.6	17.6 ± 0.6
QTc interval (ms)	49 ± 2	51 ± 2

Data is mean ± SEM. * denotes P < 0.01 by Student's t-test.

Online Supplement References

[1] Seymour AM, Chatham JC. The effects of hypertrophy and diabetes on cardiac pyruvate dehydrogenase activity. J Mol Cell Cardiol. 1997;29:2771-8.
[2] Karlstadt A, Fliegner D, Kararigas G, Ruderisch HS, Regitz-Zagrosek V, Holzhutter HG. CardioNet: a human metabolic network suited for the study of cardiomyocyte metabolism. BMC Syst Biol. 2012;6:114.
[3] Shannon P, Markiel A, Ozier O, Baliga NS, Wang JT, Ramage D, et al. Cytoscape: a software environment for integrated models of biomolecular interaction networks. Genome Res. 2003;13:2498-504.
